# First Description of Hepacivirus and Pegivirus Infection in Domestic Horses in China: A Study in Guangdong Province, Heilongjiang Province and Hong Kong District

**DOI:** 10.1371/journal.pone.0155662

**Published:** 2016-05-16

**Authors:** Gang Lu, Lingshuang Sun, Tao Xu, Dong He, Zengchao Wang, Shudan Ou, Kun Jia, Liguo Yuan, Shoujun Li

**Affiliations:** 1 College of Veterinary Medicine, South China Agricultural University, Guangzhou, P. R. China; 2 Key Laboratory of Comprehensive Prevention and Control for Severe Clinical Animal Diseases of Guangdong Province, Guangzhou, P. R. China; Oklahoma State University, UNITED STATES

## Abstract

Since 2012, three viruses, known as equine hepacivirus (EqHV), equine pegivirus (EPgV) and Theiler’s disease-associated virus (TDAV), have been discovered in equines. Given that these viruses are the newest members of the *Flaviviridae* family, genomic information concerning circulating EqHV, EPgV and TDAV strains around the world is limited. To date, no genetic surveillance studies have been performed on these three viruses in the equine population of China. Here, a total of 177 serum samples were collected from equines across China between 2014 and 2015. Using PCR, we detected viral RNA in the serum samples, six of which were EqHV positive and two of which were EPgV positive. Co-infection with the two viruses was not observed among the Chinese equines studied, and TDAV RNA was not detected in the equine serum samples collected for this study. Phylogenetic analysis of partial NS5B open reading frame (ORF), NS3 ORF, and 5’ untranslated region nucleotide sequences from EqHV as well as partial NS3 ORF sequence from EPgV indicated that EqHV and EPgV have evolved into two main clades by themselves, both of which are circulating in China. Based on the partial NS5B and NS3 ORF sequences of EqHV, the sequences of one clade were also split into two subclades. This study enriches our knowledge of the geographic distribution of these three equine viruses.

## Introduction

Hepatitis C virus (HCV) is classified as belonging to the *Hepacivirus* genus of the *Flaviviridae* family. Humans had long been considered the only true natural hosts of HCV[1–4]. Recently, novel hepaciviruses genetically related to HCV have been described among non-human hosts [1, 3, 4]. These novel viruses have no official nomenclature approved by International Committee on Taxonomy of Viruses (ICTV). Hepacivirus in non-human hosts was first described among kennel dogs with respiratory disease in the United States in 2011[2]. In subsequent studies, hepacivirus was also discovered in Old World non-human primates, equines, bats, rodents and bovines[5–9]. Among these, a horse population infected with equine hepacivirus (EqHV) was first reported in the United States in 2012[5]. Based on the luciferase immunoprecipitation system assay, 35.0% (36/103) of equine serum samples were identified positive for hepaciviruses-specific antibodies. In addition, 7.8% (8/103) of samples were found to contain EqHV RNA by reverse transcription (RT)-PCR. To date, equines with positive sera for EqHV RNA by RT-PCR have also been identified in five other countries in Europe (the United Kingdom, Germany and Hungary), South America (Brazil) and Asia (Japan)[10–15], showing prevalences of 0.7% (3/427) to 2.5% (7/210), 8.3% (25/300) to 13.4% (27/202) and 13.7% (62/453) to 22.6% (7/31), respectively.

In addition to the *Hepacivirus*, *Pestivirus* and *Flavivirus* genera, the family *Flaviviridae* also includes the genus *Pegivirus*. At first, pegivruses were found only in humans and New World monkeys [16, 17]. Since 2010, equine pegivirus (EPgV), bat pegivirus and rodent pegivirus have been discovered in the corresponding non-primate mammalian species over a wide area, including America, Europe and Asia [6, 18, 19]. Using RT-PCR with degenerate primers, equine infection with EPgV was first described in horses with elevated liver enzyme levels in the United States in 2013, with a prevalence of 14.8% (4/17) to 31.5% (6/19) between 2008 and 2012[18]. Genetic characterization of the genome of this virus revealed that it is most closely related to human pegivirus and was thus named EPgV. EPgV RNA-positive equine sera by RT-PCR have also been identified in Europe (the United Kingdom and France) and South America (Brazil)[12, 20], with prevalences of 2.8% (12/428) and 0.8% (1/114), respectively.

Theiler’s disease-associated virus (TDAV) is also classified as belonging to the *Pegivirus* genus of the *Flaviviridae* family. This virus was first reported in an outbreak of Theiler’s disease (also known as idiopathic acute hepatitis, serum-associated hepatitis or postvaccination hepatitis) in horses in the United States in 2013[21]. Massively parallel sequencing of libraries from sick horses identified a previously undescribed virus that clustered with GB viruses by phylogenetic analysis. It is possible that this virus is the causative agent of Theiler’s disease. However, since its first description, no further instances of TDAV have been reported in any equine population worldwide[12, 20].

All of these three viruses are classified as being in the *Flaviviridae* family, and they encode RNA polymerases with low fidelity, likely favoring rapid evolution[22]. Although it has only been known for no more than 30 years, HCV has evolved into 7 distinct genotypes and 67 subtypes[22, 23]. Human pegivirus was first discovered in 1995, and now, six genotypes of this virus have been confirmed by phylogenetic analysis[24]. In addition, three highly diverse clades were identified in both bat pegivirus and hepacivirus[6]. To date, genomic information concerning circulating virus strains of EqHV, EPgV and TDAV around the world is limited. In particular, the extent of EqHV, EPgV and TDAV infection in the equine population of China remains unclear. Considering these facts, in the current study, we investigated the molecular prevalence and genetic diversity of EqHV, EPgV and TDAV in China based on molecular research, with the aim of elucidating the geographic distribution and genetic evolution of these three equine viruses.

## Materials and Methods

### Samples

Between 2014 and 2015, a total of 177 serum samples were collected from 163 horses (*Equus caballus*), 8 mules (*Equus mulus*) and 6 donkeys (*Equus asinus*) in China. The samples came from work equines in Hailun City, Heilongjiang Province, Northern China, and racehorses in Guangzhou, Huizhou, and Shenzhen Cities, Guangdong Province, and in Hong Kong, southern China (Table 1). The serum samples were stored at -70°C immediately after collection.

**Table 1:** Detailed information about collection period, geographical origin, species and number of equine samples used for molecular research hepaci- and pegiviruses in China during 2014–2015.

Collection period	Collection origin	Species	For work or race	Number	EqHV positive	EPgV positive	TDAV positive
October, 2014	Hailun	horse	work	19	1/19(5.3%)	0/19(0.0%)	0/19(0.0%)
		mule	work	8	0/8(0.0%)	0/8(0.0%)	0/8(0.0%)
		donkey	work	6	0/6(0.0%)	0/6(0.0%)	0/6(0.0%)
June, 2014-September, 2015	Guangzhou	horse	race	67	2/67(3.0%)	0/67(0.0%)	0/67(0.0%)
October,2014-October, 2015	Huizhou	horse	race	44	2/44(4.5%)	0/44(0.0%)	0/44(0.0%)
December, 2014	Shenzhen	horse	race	14	0/14(0.0%)	2/14(14.3%)	0/14(0.0%)
2014	Hong Kong	horse	race	19	1/19(5.3%)	0/19(0.0%)	0/19(0.0%)
Total				177	6/177(3.4%)	2/177(1.1%)	0/177(0.0%)

### Ethical considerations

We obtained permission to use the animals’ sera in this study from all owners of the equines. The serum samples used in this study were collected with the assistance of local veterinarians. The serum sample collection method was conducted under the guidance of the South China Agricultural University Experimental Animal Welfare Ethics Committee. All procedures associated with the animal experiments were also approved by the South China Agricultural University Experimental Animal Welfare Ethics Committee (reference number 2014–05).

### RT-PCR and sequencing

Total RNA was extracted from 200 μL equine serum using RNAiso Plus (Takara, Dalian) according to the manufacturer’s protocol. The resulting RNA was eluted in a final volume of 20 μL using nuclease-free water. A total of 2 μL of diluted RNA was used as a template for RT into cDNA using the GoScript™ Reverse Transcription System (Promega, Shanghai, China), with a mixture of oligo(dT)15 and random primers as the RT primers. The synthesized cDNA was then subjected to PCR using PrimeSTAR® HS (premix) (Takara, Dalian, China).

To determine whether the equine serum samples contained EqHV RNA, partial sequence of the NS5B open reading frame (ORF) sequence was amplified by nested PCR using two sets of degenerate primers, as described previously[11] (Table 2). The primers were slightly modified in this study based on newly published sequence data for EqHV. For the first round, 2 μL of cDNA was used as the template along with the primers NS5O-F and NS5O-R (each primer: 0.4 uM). PCR was performed using the following cycling conditions: 35 cycles at 98°C for 10 s, 50°C for 15 s, and 72°C for 30 s, followed by 1 cycle at 72°C for 5 min. For the second round, 2 μL of the first PCR product was used as the template along with the primers NS5I-F and NS5I-R (each primer: 0.4 uM). The PCR product from the second round was electrophoresed on a 1.5% agarose gel. Serum samples that yielded PCR products of approximately 300 bp were considered to be positive. All the samples were also subjected to nested PCR with the primers NS3O-F, R and NS3I-F, R (each primer: 0.4 uM), targeting the NS3 ORF sequence, as well as with the primers NS5UO-F, R and NS5UI-F, R (each primer: 0.4 uM), targeting the 5’ untranslated region (UTR) of EqHV. Serum samples that yielded PCR products of approximately 200 bp and 250bp were considered to be positive. Blunt-ended PCR products were cloned into the PLB vector using the Lethal Based Fast Cloning Kit (TianGen, Beijing, China) and were then sequenced (BGI, Guangdong, China). Sequencing was performed in 3730xl DNA Analyzer (Applied Biosystems, Guangdong, China), using BigDye^TM^ Terminator V3.1 Cycle Sequencing Kit (Applied Biosystems, Guangdong, China).

**Table 2:** The primer sequences for EqHV and EPgV in our study.

The amplified virus	Primer name	Primer sequence (primer sense: 5 '- 3')
EqHV	NS5O-F	ARTGYTTTGACTCBACBGTCACTC
	NS5O-R	RCTRTGACTRATYRTYTCCCAACTCG
	NS5I-F	CAYGAYRTAGAHACTGAGAGRGA
	NS5I-R	TCRTCTTCCTCMRCGCCYTTRCTGG
	NS3O-F	ATHTGTGATGARTGCCAYAGYAC
	NS3O-R	TAGTAGGTBACAGCRTTAGCYCC
	NS3I-F	TCYAARGGTGTDAAGCTTGTTGT
	NS3I-R	TGRCARAAGYTAAGRTGYCTYCC
	NS5UO-F	ACAYYACCATGTGTCACTCCCCCT
	NS5UO-R	CYCATGTCCTATGGTCTACGAGA
	NS5UI-F	ACACGGAAAYRRGTTAACCAYACYC
	NS5UI-R	GCCCTCGCAAGCATCCTATCAG
EPgV	TDAV-3744F	GGAGCCCGGAGCGCATGGGTA
	TDAV-4098R	TGGCAGGGACAAGGGTGGACT

To investigate the frequency of EPgV and TDAV infection in equines, a conserved region of the NS3 ORF sequence from these two viruses was amplified by PCR using the same procedure as for EqHV and the primers TDAV-3744F and 4098R (each primer: 0.4 uM) (Table 2)[25]. For the second PCR round, 2 μL of the PCR product from the first round was used as the template, and the same primer pair was used again. Serum samples yielding PCR products of approximately 350 bp by agarose gel electrophoresis were considered positive.

**Fig 1:**
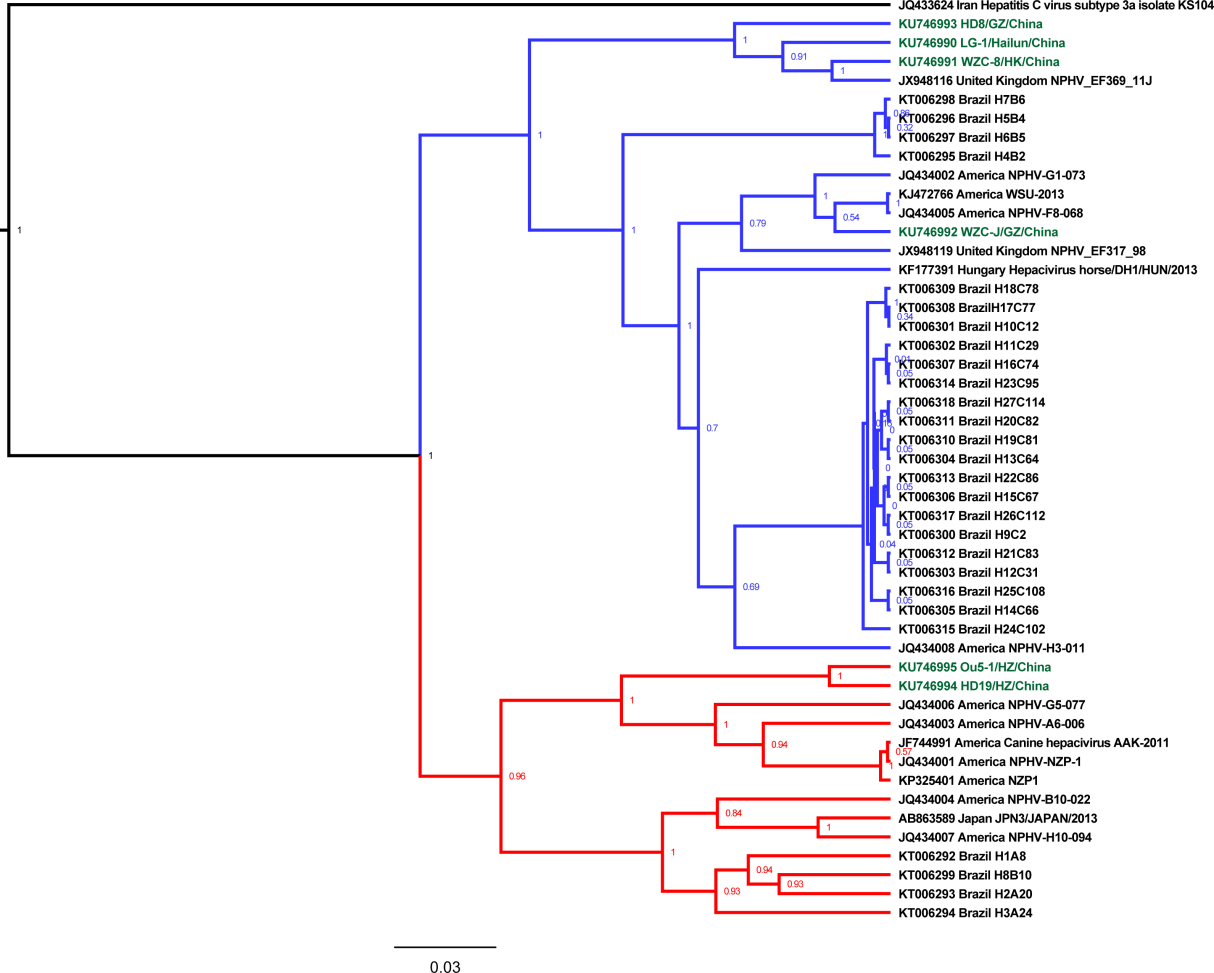
Phylogenetic analysis of the partial NS5B ORF sequences from EqHV. The field strains from China are indicated in green. Two distinct lineages for clades 1 and 2 are shown in blue and red, respectively. Details of the strain names, GenBank numbers and country names are indicated in the phylogenetic tree.

**Fig 2:**
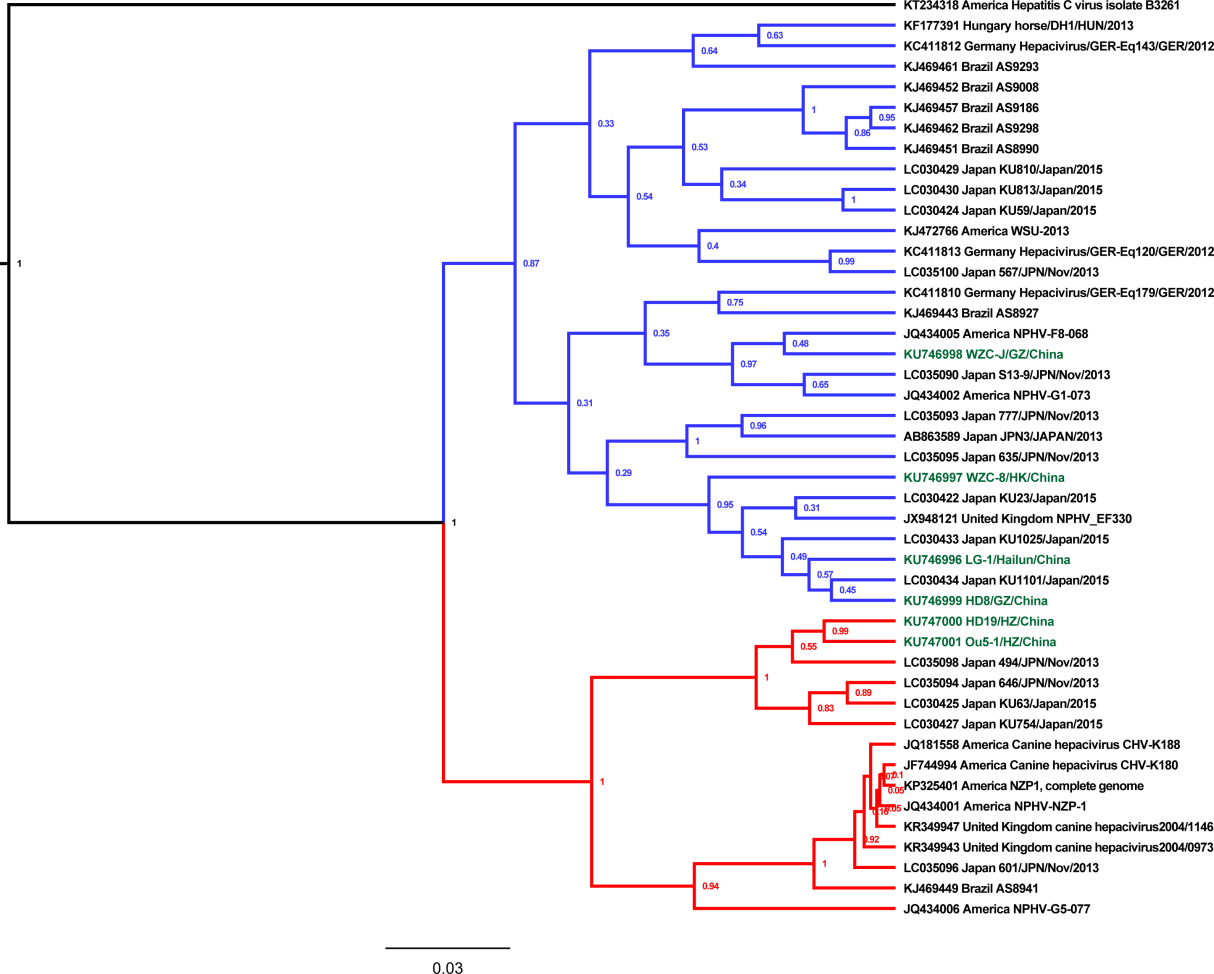
Phylogenetic analysis of the partial NS3 ORF sequences from EqHV. The analysis method has been described in Fig 1.

**Fig 3:**
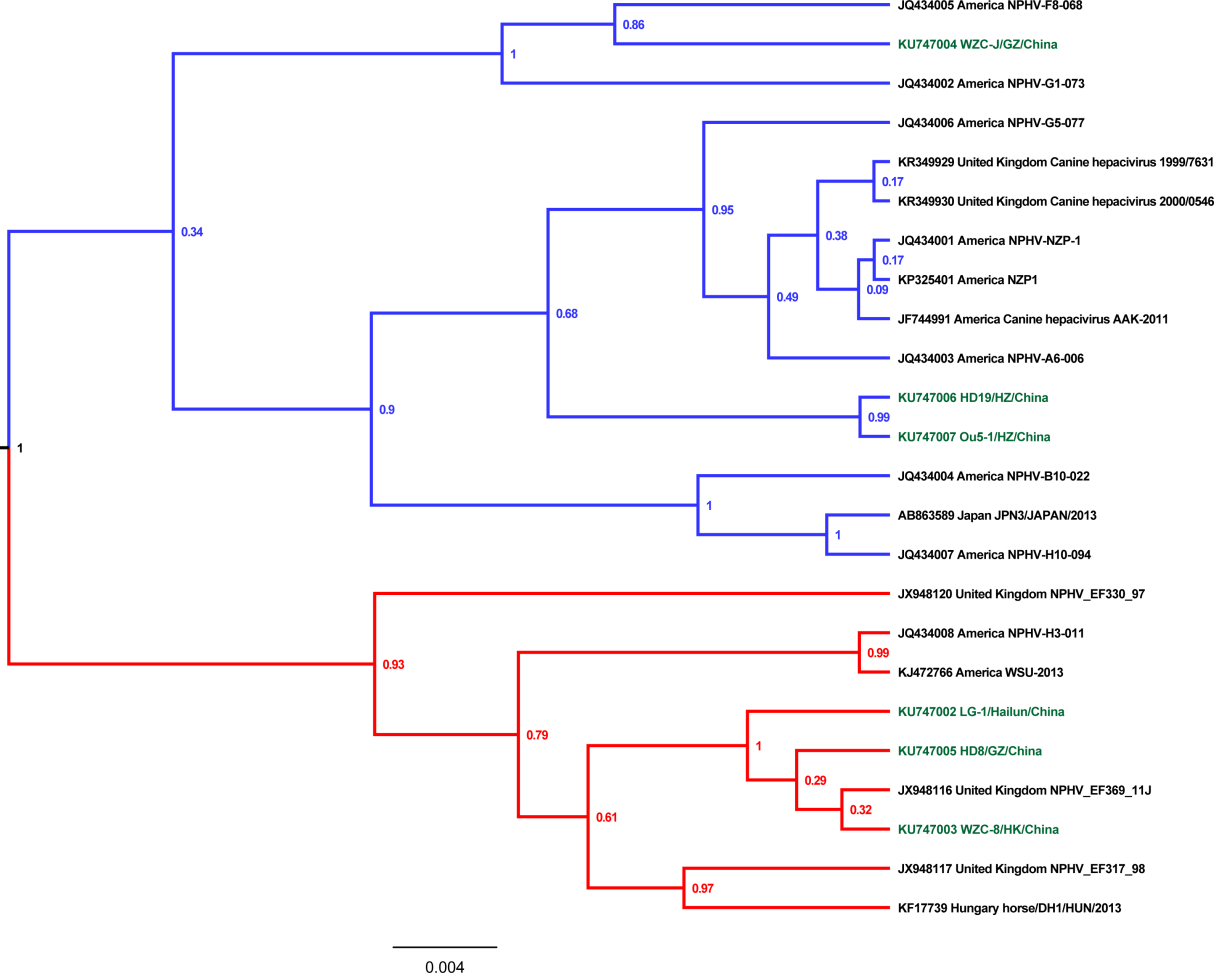
Phylogenetic analysis of the partial 5’UTR nucleotide sequences from EqHV. The analysis method has been described in Fig 1.

**Fig 4:**
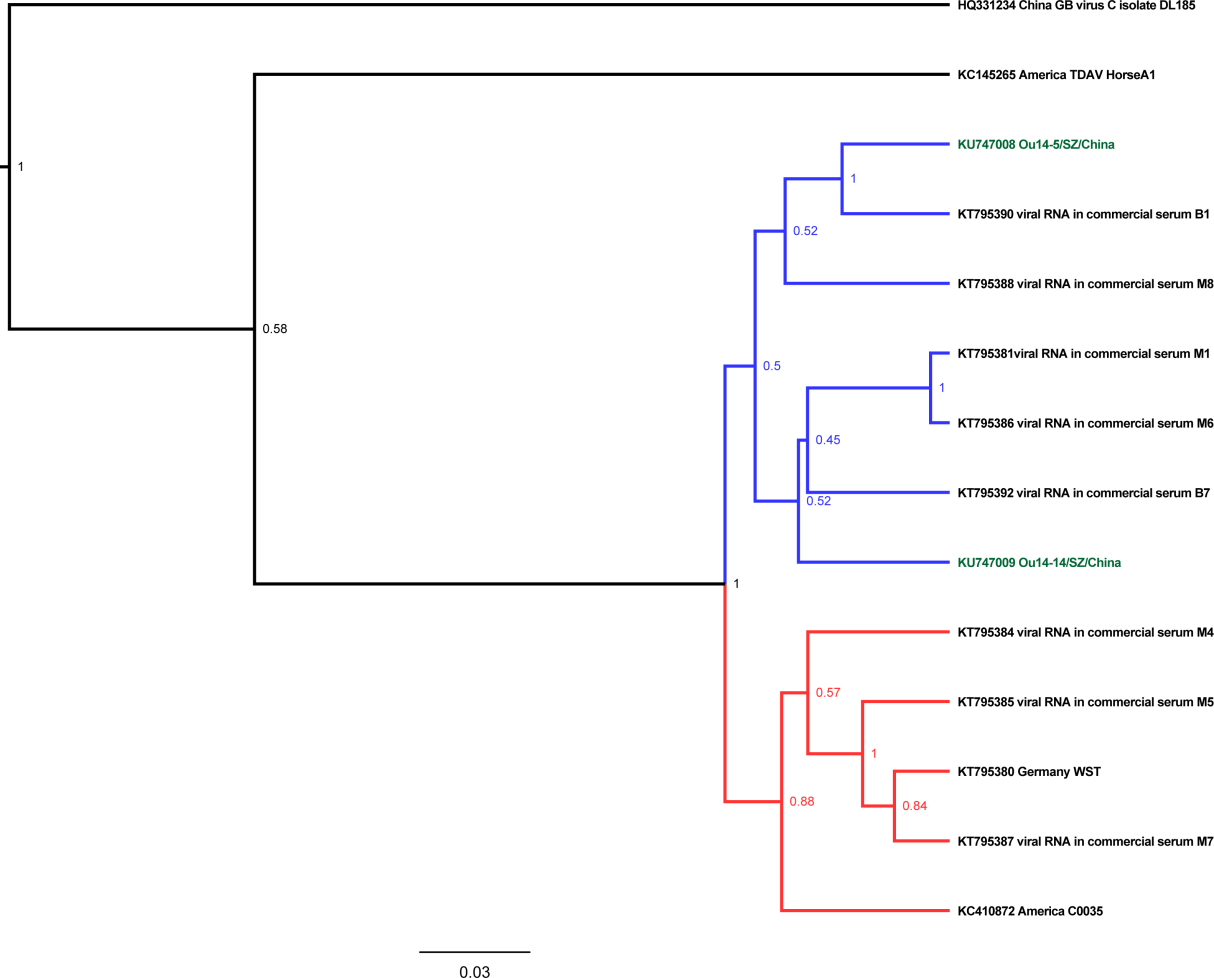
Phylogenetic analysis of the partial NS3 ORF sequences from EPgV. The analysis method has been described in Fig 1.

The viral sequences have been submitted to the GenBank database (listed in S1 Appendix). Details concerning strain names and the corresponding GenBank numbers are shown in Figs 1–4.

### Genetic and phylogenetic analyses

To perform evolutionary analyses of EqHV, EPgV and TDAV circulating in China, the nucleotide sequences of the viruses available in GenBank (http://www.ncbi.nlm.nih.gov/) were used to construct phylogenetic trees. Using the BioEdit 7.0.9.0 software program, the nucleotide sequences of the field strains from China were edited and aligned with those from other countries, and the nucleotide similarity values between the sequences were calculated. The details of the nucleotide similarity values between the sequences were listed in S2 Appendix. Finally, a maximum clade credibility (MCC) phylogenetic tree based on an HKY substitution model was inferred using the Beast v1.8.2 software program (Figs 1–4). Posterior probability values are indicated at the nodes.

## Results

### Identification of viral RNA in equine sera

A total of 177 serum samples were collected from equines in China from 2014–2015, of which 33 samples came from work equines in northern China, with the remainder coming from racehorses in southern China (Table 1). Detection of EqHV RNA in the equine sera was performed using nested PCR targeting portions of the NS3 and NS5B ORF sequences and the viral 5’UTR. In total, 3.4% (6/177) of the serum samples were identified as positive for EqHV RNA using nested RT-PCR and sequencing. One of the six positive samples was from a work horse, with the other five coming from racehorses. The six field EqHV strains were named LG-1/Hailun/China (from Hailun City), WZC-8/HK/China (from Hong Kong), WZC-J/GZ/China and HD8/GZ/China (from Guangzhou City), and HD19/HZ/China and Ou5-1/HZ/China (from Huizhou City). No EqHV RNA was detected in the samples collected from Shenzhen City. In addition, the samples collected from the mules and donkeys were negative for EqHV RNA.

For EPgV and TDAV, two rounds of PCR were performed using the same set of primers to detect conserved regions in both viruses. Two samples from racehorses in Shenzhen City were found to be positive by nested RT-PCR. At least five positive *Escherichia coli* clones per sample were collected for sequencing, and BLAST hits for all clones matched the nucleotide sequence of EPgV. The field EPgV strains were named Ou14-5/SZ/China and Ou14-14/SZ/China. For EPgV, the overall prevalence among the samples was found to be 1.1% (2/177), which was lower than that for EqHV. Similar to what was observed for EqHV, the serum samples from the mules and donkeys were negative for EPgV RNA. For TDAV, no viral RNA was detected in the equine serum samples collected in this study.

### Genetic and phylogenetic characterization of the viral sequences

To perform genetic and phylogenetic characterizations of the six EqHV strains obtained in China, a total of 43 partial NS5B sequences of hepacivirus were obtained from the GenBank database, including 43 sequences from EqHV, 1 sequence from canine hepacivirus and 1 sequence from HCV(Fig 1). The nucleotide similarity among the NS5B ORF sequence from the field horse EqHV strains in China varied from 82.14% (between Ou5-1/HZ/China and WZC-J/GZ/China) to 96.1% (between Ou5-1/HZ/China and HD19/HZ/China). When comparing the NS5B ORF sequence of EqHV between the Chinese strains and the 43 strains from other countries, the nucleotide similarity varied from 77.68% (between Ou5-1/HZ/China and the Brazilian strain H24C102) to 96.42% (between WZC-J/GZ/China and the American strain NPHV-G1-073). The phylogenetic analysis revealed that the NS5B ORF sequence of HCV clustered as an outgroup with EqHV, whereas the NS5B ORF sequence of canine hepacivirus clustered with the NS5B ORF sequence of EqHV. The NS5B ORF sequence of EqHV has evolved into two main distinct clades, as indicated by phylogenetic analysis, which we designate as clade 1 and clade 2. We found that the NS5B sequences of LG-1/Hailun/China, WZC-8/HK/China, HD8/GZ/China and WZC-J/GZ/China clustered together within clade 1, whereas the NS5B sequences of HD19/HZ/China and Ou5-1/HZ/China, both identified from horse sera in Huizhou City, clustered in clade 2. This evidence indicates that EqHV strains from both clades are co-circulating within the equine population of China. This phenomenon can also be observed in America and Brazil. Although clustered within the same clade, the NS5B ORF sequence of the strain identified in northern China (LG-1/Hailun/China) was most closely related to WZC-8/HK/China and HD8/GZ/China relative to WZC-J/GZ/China from southern China, with nucleotide similarities of 96.1%, 92.85% and 86.68%, respectively. Based on the phylogenetic tree, it was also found that the NS5B ORF sequence of WZC-8/HK/China, HD8/GZ/China, and WZC-J/GZ/China grouped together in a subclade within clade 1, along with one strain from the United Kingdom. The strain WZC-J/GZ/China grouped within another subclade of clade 1, along with 29 strains from the United Kingdom, Hungary, America and Brazil.

To determine the evolutionary model of the NS3 ORF and the 5’UTR sequence of EqHV in China, nucleotide sequences from 39 (1 from human, 3 from canine, 25 from horse) and 18 (3 from canine, 16 from horse) hepacivirus strains were selected from the GenBank database. The nucleotide identity ranged from 84.97% (between Ou5-1/HZ/China and WZC-J/GZ/China) to 94.79% (between Ou5-1/HZ/China and HD19/HZ/China) for the NS3 ORF sequence between the Chinese field strains. For the 5’UTR, the nucleotide identity ranged from 94.87% (between WZC-8/HK/China and WZC-J/GZ/China) to 100% (between Ou5-1/HZ/China and HD19/HZ/China). When comparing the NS3 ORF and the 5’UTR sequences of EqHV between China and other countries, the nucleotide similarity ranged from 82.65% (between Ou5-1/HZ/China and the American strain NPHV-G1-073) to 95.95% (between HD8/GZ/China and the Japanese strain KU1101/Japan/2015) and from 93.58% (between WZC-8/HK/China and the United Kingdom strain NPHV-EF317-98) to 99.57% (between WZC-8/HK/China, LG-1/Hailun/China and NPHV-EF369-11J), respectively. Phylogenetic characterization indicated that the NS3 ORF and 5’UTR sequences of EqHV grouped into two clades, and both clades were found in the horse populations of China, America and the United Kingdom (Fig 2, Fig 3). For the NS3 ORF, it was observed that HD19/HZ/China and Ou5-1/HZ/China clustered into one clade, whereas the other four Chinese strains grouped into another clade. For the 5’UTR, we found that HD19/HZ/China, Ou5-1/HZ/China and WZC-J/GZ/China clustered together in one clade, with the other three Chinese strains forming another.

The BLAST results for the partial NS3 ORF sequence from the two EPgV strains (Ou14-5/SZ/China and Ou14-14/SZ/China) in China aligned with two other EPgV strains: the WST strain from Germany and the C0035 strain from America. In addition, the hits included nucleotide sequences from EPgV that were identified in the commercial equine serum for use in cell culture. The nucleotide similarity between Ou14-5/SZ/China and Ou14-14/SZ/China was 93.52%. The nucleotide identities between Ou14-5/SZ/China and WST and between Ou14-5/SZ/China and C0035 were 91.31% and 92.02%, respectively. In addition, the nucleotide identities between Ou14-14/SZ/China and WST and between Ou14-14/SZ/China and C0035 were 90.99% and 92.59%, respectively. A phylogenetic tree was constructed using the partial NS3 ORF sequence from 1 GB virus C strain, 1 TDAV strain, 4 EPgV strains and 8 EPgV sequences obtained from commercial equine serum (Fig 4). The results indicated that GB virus C and the TDAV strain clustered as an outgroup. Moreover, similar to what was observed for EqHV, the NS3 ORF sequence of EPgV divided into two clades. The two Chinese strains were grouped into the same clade, and the WST strain from Germany and the C0035 strain from America formed another clade. The EPgV sequences from the commercial equine serum were found in both clades.

## Discussion

Very recently, three novel viruses of EqHV, EPgV and TDAV belonging to the *Flaviviridae* family were identified in equines[1, 4, 5, 18, 21]. To investigate the infection frequency of these three viruses in the equine population of China, a total of 177 serum samples were collected from three regions: Guangdong Province, Heilongjiang Province and Hong Kong District, and were tested by nested RT-PCR protocols for detection of horse hepatic- and pegiviral RNA. The prevalences of positive equine sera for EqHV RNA were found to be 3.4% in China (Table 1), which were lower than the prevalences reported in Brazil and Japan[10, 14, 15] and slightly higher than in European countries[11–13]. The prevalences of positive equine sera for EPgV RNA were found to be 1.1% in China(Table 1), which were lower than the rates identified in European countries and America and slightly higher than in Brazil[12, 18, 20]. Co-infection with both EqHV and EPgV was not observed in our study. We note that no samples collected from donkeys or mules were positive for EqHV or EPgV, consistent with previous studies that detected viral RNA or specific antibodies in serum samples[10–12]. However, considering the smaller sample number of donkeys and mules (n = 14) compared with horses (n = 163) in this study, whether these two species in China can be infected with EqHV still needs further study. Since the first description of TDAV, no TDAV-positive equine serum samples have been found in other studies[12, 20], including this one, although TDAV RNA contamination was confirmed in horse sera used for cell culture propagation in Chile, Germany and Italy[25]. Until now, most of the studies on TDAV in the equine population were estimated by detecting the viral RNA in the clinical samples (serum, plasma) of animals. The true prevalence of TDAV is still poorly investigated and broad seroepidemiological studies are needed in the equine population.

Within host cells, these viruses propagate through error-prone RNA replication, and the pegiviruses and hepaciviruses are regarded as highly genetically variable[22]. In our study, phylogenetic analysis indicated the presence of two main clades when analyzing partial NS5B ORF, NS3 ORF, and 5’UTR nucleotide sequences from EqHV as well as partial NS3 ORF nucleotide sequences from EPgV (Figs 1–4). It was noted that two clades of EqHV were co-circulating in the equine population in China, America and Brazil when subjected to the partial NS5B and NS3 ORF nucleotide sequences (Fig 1, Fig 2). We found that the sequences of one clade were also split into two subclades based on the partial NS5B and NS3 ORF nucleotide sequences of EqHV. Considering the partial NS5B ORF nucleotide sequences from clade 1 of EqHV, the four strains WZC-8/HK/China, HD8/GZ/China, WZC-J/GZ/China and NPHV-EF369-11J were clustered together in one subclade, whereas the other 30 strains were clustered as another subclade.

The route of transmission for EqHV has not been fully described. However, adult horses and foals that were directly inoculated with plasma containing EqHV could be infected by the virus and developed acute and chronic liver disease detected by liver-specific enzymes and/or by histopathology[26]. To date, no studies on the transmission pattern of EPgV have been reported. As for TDAV, this virus has been regarded as the possible infectious agent associated with Theiler’s disease. It was found that the virus can be transmitted between horses by experimental inoculation[21, 27]. Accordingly, considering the potential threat to the horse health, it is recommended to detect the EqHV and TDAV RNA in the blood or the blood product, before performing blood transfusion or treated horse with equine blood product.

Genetic analysis of hepacivirus sequences from different mammalian species showed that EqHV clusters into a single group with canine hepacivirus[3, 10]. It has been hypothesized that EqHV jumped the species barrier to infect dogs in approximately 1970[3]. Although no direct evidence of hepacivirus transmission between horses and humans was found in one recent study[28], considering the high mutation rate of hepaciviruses, the history of possible cross-transmission to dogs, and its genetic similarity to HCV, continued genetic surveillance of EqHV is still necessary.

Our study is the first to describe EqHV and EPgV infection in the equine population of China. Phylogenetic analysis involving partial NS5B ORF, NS3 ORF, and 5’UTR nucleotide sequences from EqHV as well as partial NS3 ORF nucleotide sequences from EPgV revealed that these viruses have evolved into two main clades, with both clades of EqHV currently circulating in China. One limitation of our study was that the equine serum sample size was relatively small and the samples were collected only three regions in China: Guangdong Province, Heilongjiang Province and Hong Kong District. The equine serum sample may not have been fully representative of horses in China. Thus, further investigations are required to confirm the circulation of EqHV, EPgV and TDAV in China.

## Supporting Information

S1 Appendix The viral sequences identified in our study. (XLS)

S2 Appendix The nucleotide similarity values between sequences of different EqHV and EPgV strains. (GB)
